# Pretomanid with bedaquiline and linezolid for drug-resistant TB: a comparison of prospective cohorts

**DOI:** 10.5588/ijtld.21.0035

**Published:** 2021-06-01

**Authors:** S. Oelofse, A. Esmail, A. H. Diacon, F. Conradie, O. Olayanju, N. Ngubane, P. Howell, D. Everitt, A. M. Crook, C. M. Mendel, G. H. Wills, M. Olugbosi, A. del Parigi, E. Sun, A. Calatroni, M. Spigelman, K. Dheda

**Affiliations:** 1 Centre for Lung Infection and Immunity, Division of Pulmonology and UCT Lung Institute, Department of Medicine, University of Cape Town (UCT), Cape Town, South Africa; 2 Task Applied Science and Stellenbosch University, Cape Town, South Africa; 3 Clinical HIV Research Unit, Faculty of Health Sciences, University of Witwatersrand, Johannesburg, South Africa; 4 Sizwe Tropical Disease Hospital, Sandringham, South Africa; 5 King DinuZulu Hospital Complex, Durban, South Africa; 6 TB Alliance, New York, NY, USA; 7 Institute of Clinical Trials and Methodology, University College London, London, UK; 8 TB Alliance, Pretoria, South Africa; 9 Institute of Infectious Diseases and Molecular Medicine, University of Cape Town, Cape Town, South Africa; 10 Faculty of Infectious and Tropical Diseases, London School of Hygiene & Tropical Medicine, London, UK; 11 Rho Federal Systems Division, Inc., Durham, NC, USA

**Keywords:** drug resistance, tuberculosis, drug treatment, BPaL, bedaquiline, linezolid

## Abstract

**BACKGROUND:**

There are no data comparing the 6–9 month oral three-drug Nix regimen (bedaquiline, pretomanid and linezolid [BPaL]) to conventional regimens containing bedaquiline (B, BDQ) and linezolid (L, LZD).

**METHODS:**

Six-month post end-of-treatment outcomes were compared between Nix-TB (*n* = 109) and 102 prospectively recruited extensively drug-resistant TB patients who received an ~18-month BDQ-based regimen (median of 8 drugs). A subset of patients received BDQ and LZD (*n* = 86), and a subgroup of these (*n* = 75) served as individually matched controls in a pairwise comparison to determine differences in regimen efficacy.

**RESULTS:**

Favourable outcomes (%) were significantly better with BPaL than with the B–L-based combination regimen (98/109, 89.9% vs. 56/86, 65.1%; adjusted relative risk ratio [aRRR] 1.35; *P* < 0.001) and in the matched pairwise analysis (67/75, 89.3% vs. 48/75, 64.0%; aRRR 1.39; *P* = 0.001), despite significantly higher baseline bacterial load and prior second-line drug exposure in the BPaL cohort. Time to culture conversion (*P* < 0.001), time to unfavourable outcome (*P* < 0.01) and time to death (*P* < 0.03) were significantly better or lower with BPaL than the B-L-based combinations.

**CONCLUSION:**

The BPaL regimen (and hence substitution of multiple other drugs by pretomanid and/or higher starting-dose LZD) may improve outcomes in drug-resistant TB patients with poor prognostic features. However, prospective controlled studies are required to definitively answer this question.

## Abstract (FR)

**CONTEXTE:**

Il n’y a pas de données comparant les protocoles oraux de 6–9 mois associant trois médicaments (bédaquiline, prétomanide et linézolide [BPaL]) aux protocoles conventionnels contenant de la bédaquiline (B, BDQ) et du linézolide (L, LZD).

**MÉTHODE:**

On a comparé les résultats 6 mois après la fin du traitement entre Nix-TB (*n* = 109) et 102 patients TB recrutés prospectivement qui ont reçu un protocole de BDQ d’environ 18 mois (médiane de 8 médicaments). Un sous ensemble de patients a reçu de la BDQ et du LZD (*n* = 86) et un sous-groupe de ces derniers (*n* = 75) a servi de témoins appariés individuellement pour des comparaisons par paires afin de déterminer les différences d’efficacité du protocole.

**RÉSULTATS:**

Des résultats favorables (%) ont été significativement meilleurs avec BPaL qu’avec le protocole combiné basé sur B-L (98/109, 89,9% contre 56/86, 65,1% ; rapport de risque relatif ajusté [RRRa] 1,35 ; *P* < 0,001) et dans l’analyse par paires (67/75, 89,3% contre 48/75, 64,0% ; RRRa 1,39 ; *P* = 0,001) malgré une charge bactérienne initiale significativement plus élevée et une exposition préalable aux médicaments de deuxième ligne. Le délai de conversion de la culture (*P* < 0,001), le délai de résultats défavorables (*P* < 0,01) et de décès (*P* < 0,03) ont été significativement meilleurs ou plus bas avec BPaL comparés à la combinaison à base de BL.

**CONCLUSION:**

Le protocole BPaL (et donc la substitution de multiples autres médicaments par le prétomanide et/une dose de départ plus élevée de LZD) pourrait améliorer les résultats des patients TB résistante ayant des facteurs de pronostic négatifs. Des études prospectives contrôlées sont cependant requises pour répondre définitivement à cette question.

TB IS THE FOREMOST INFECTIOUS DISEASE globally and almost a billion people have died due to TB over the past two centuries.^1^ Control efforts in several parts of the world have been subverted by the growing incidence of multidrug-resistant TB (MDRTB) and extensively drug-resistant TB (XDR-TB).^2^ The global burden of MDR- and XDR-TB taken together in 2018 was estimated at ∼half a million cases and the number of confirmed cases has nearly doubled in the last 5 years.^2^,^3^ The 2019 WHO Global TB Report estimated favourable outcome rates for MDR-TB to be ~56%, and drug-resistant TB (DRTB) as a whole accounted for ~25% of global TB mortality.^3^ The introduction of novel and repurposed drugs such as bedaquiline (BDQ) and linezolid (LZD) appears to improve mortality and outcomes.^4^–^10^ However, even with the use of these newer agents, unfavourable outcome rates in MDR-TB remain at ~25% and at ~35% for XDR-TB.^4^–^6^ Treatment options therefore remain limited. It should be noted that only one in three cases of DR-TB in 2018 received treatment.^3^ Furthermore, improved outcomes were only achieved in selected studies and regions, while the unfavourable outcome rate in 2018 globally remained dismal, at ~45% for MDR-TB and at ~60% for XDR-TB.^3^ Thus, there is an urgent need for alternative and improved regimens, particularly for XDR-TB. Although the optimal duration of a specific drug combination or treatment regimen for DR-TB remains unclear, a longer duration of treatment (18–20 months) and higher pill burden has been associated with poor adherence.^2^ Hence, there is a pressing need for shorter regimens with increased effectiveness and reduced pill burden. This is especially so in patients with poorer prognosis, including those with XDR-TB and treatment-intolerant or failed MDR-TB.

Pretomanid (Pa, PTM), a nitro-imidazooxazine that blocks mycobacterial cell wall production and increases nitric oxide release, was recently approved by the US Food and Drug Administration (US FDA) as part of a combination regimen BPaL (with BDQ and LZD) for the treatment of adults with pulmonary XDR-TB, or treatment-intolerant or non-responsive MDR-TB. This was based in part on a 90% treatment success rate in the first 107 patients followed to the primary endpoint in a Phase 3 single-arm clinical study – known as the Nix-TB trial.^11^ Preclinical data highlighted the individual contribution of all three drugs (BDQ, PTM and LZD) in achieving the efficacy of the three-drug regimen.^12^ Furthermore, PTM demonstrated dose-related antimycobacterial activity in a 2-week monotherapy clinical study in drug-susceptible TB patients.^13^ However, Nix-TB was an open-label study without a control arm. Thus, to date there are no clinical data comparing the efficacy of the BPaL regimen to BDQ and LZD-based (B–L) regimens containing companion drugs but without PTM. To address this question, we compared outcomes from the Nix-TB cohort to a prospectively recruited clinical registry-based cohort of patients with XDR-TB from Cape Town, South Africa.^4^

## MATERIALS AND METHODS

### Study design and participants

A cohort-comparison study with prospective follow-up was performed post-hoc between two cohorts: the Nix-TB cohort (BPaL population) and the XDR-TB cohort (BDQ- and mostly LZD-treated population with companion drugs). The Nix-TB cohort consisted of 109 patients enrolled in a prospective intervention trial between April 2015 and November 2017 from three study sites in South Africa: Sizwe Tropical Disease Hospital, Johannesburg; Task Applied Science at Brooklyn Chest Hospital, Cape Town; and King DinuZulu Hospital Complex in Durban, South Africa. After obtaining informed consent, the comparator cohort was enrolled between August 2013 and August 2018 in a prospectively recruited observational trial from Brooklyn Chest Hospital, which is the designated XDR-TB treatment centrein the Western CapeProvinceof South Africa. Treatment was directly observed by trained health workers (details have been published).^4^ Patients from both cohorts were treated in a hospital setting for a variable duration of their treatment and had microbiologically confirmed TB with isolates showing MDR-TB or XDR-TB.

Demographic and clinical information was obtained by trained healthcare workers. BPaL cohort patients were actively monitored, and their data collected in real time. For the comparator group, information was regularly extracted by a trained healthcare worker from patient records and associated healthcare and laboratory systems. The demographic variables collected included age, sex and body weight at diagnosis. The clinical variables collected included HIV status, CD4 count at baseline for those coinfected with HIV, smear status at baseline, medications used in the regimen and adverse events. Further elaboration of the cohorts and treatment-related adverse events have been published in the papers describing each cohort.^4^,^11^

Ethical approval was obtained from the relevant human research ethics committees (HRECs) for both cohorts (University of Cape Town HREC, Cape Town, South Africa; HREC reference 038/2008; and Pharma Ethics (123 Amcor Road, Lyttlelon Manor, Pretoria, South Africa; no14065930).

### Treatment regimens

The Nix-TB regimen comprised BDQ, PTM and LZD (BPaL). The first 44 participants were started on LZD 600 mg twice daily, and the remaining 65 were started on 1200 mg once daily with dosage reductions, interruptions and discontinuation allowed for LZD, depending on tolerability. The three medications were prescribed for 6 months in the majority of the population; treatment was prolonged to 9 months as per the protocol in two participants whose sputum had not culture converted to negative by Month 4 of treatment.

The comparator population received a regimen in line with the South African National TB Programme (NTP). For the selected population, this included a core comprising BDQ (100%) and LZD (84%) (at a South African standard dose of 600 mg daily). These patients also often received pyrazinamide (98%), clofazimine (96%), levofloxacin (91%), para-amino-salicylic acid (PAS) (90%) and terizidone (89%). The median number of medications prescribed in this population was 8. The aimed duration of treatment in this population was 18–24 months, with a median duration of 18 months in those who completed the regimen.

### Outcomes

The primary treatment efficacy endpoint in the Nix-TB population was defined (as per protocol) as the incidence of bacteriological failure or relapse, or clinical failure at 6 months after the end of treatment. Favourable outcomes for this analysis included patients with a negative culture status at 6 months after the end of treatment, those who had not already been classified as having an unfavourable outcome and those whose last positive culture result was followed by at least two negative culture results. Unfavourable outcomes included all patients who died, those who relapsed prior to 6 months after treatment completion (defined for this analysis as “treatment failures”) or those who were lost to follow-up (LTFU) or those who withdrew from the trial.

Treatment outcomes for the comparator population were defined according to the adapted 2013 WHO criteria as modified by Furin et al.^14^,^15^ Favourable outcomes included patients who achieved cure (defined as treatment completed without evidence of failure, with three or more consecutive negative sputum culture results after the intensive phase of treatment) or treatment completion. Unfavourable outcomes were assigned to patients who died, failed treatment, defaulted or were LTFU.^4^ The assessment time-point in the comparator population was 24 months after the start of treatment with the BDQ-based regimen (or 6 months post-end-of treatment as most patients received ~18 months of therapy).

## RESULTS

### Demographic and clinical characteristics

The baseline characteristics between the two populations are shown in Table 1, in Supplementary Table S1A for the subgroup that received both BDQ and LZD, and in Supplementary Table S1B when these participants were matched for demographic features and HIV status. Antiretroviral coverage was 100% in both cohorts.

**Table 1:** Comparison of clinical characteristics and demographic data ^*^

	Nix-TB (*n* = 109) *n/N* (%)	XDR-TB comparator (*n* = 102) *n/N* (%)	*P* value
Sex			0.12
Female	52/109 (47.7)	37/102 (36.3)	
Male	57/109 (52.3)	65/102 (63.7)	
Age at treatment initiation, years, mean ± SD	35.6 ± 10.1	36.8 ± 11.2	0.41
Weight, kg, mean ± SD	56.9 ± 15.0	53.1 ± 10.0	0.03
Baseline smear results			<0.01
Negative	31/109 (28.4)	74/102 (72.5)	
Positive	78/109 (71.6)	28/102 (27.5)	
HIV status			1.00
Negative	53/109 (48.6)	50/102 (49.0)	
Positive	56/109 (51.4)	52/102 (51.0)	
CD4 count at baseline, cells/mm^3^ median [IQR]	406 [282–632]	129 [56–283]	<0.01
Duration of second-line drug exposure prior to interventional treatment initiation, weeks			0.011
Not available	0	11	
≤8	21/109 (19.3)	32/91 (35.2)	
>8	88/109 (80.7)	59/91 (64.8)	
LZD			<0.01
No	0/109 (0.0)	16/102 (15.7)	
Yes	109/109 (100.0)	86/102 (84.3)	

^*^ Relevant demographic data comparisons for Comparison 2 (patients who received BDQ and LZD) are shown in Supplementary Table S1A and for Comparison 3 (patients who received BDQ and LZD, and who were matched for demographic characteristics and HIV status) are shown in Supplementary Table S1B.

XDR-TB = extensively drug-resistant TB; SD = standard deviation; IQR = interquartile range; LZD = linezolid; BDQ = bedaquiline.

### Treatment outcomes in the different comparison groups

#### Overall comparison (Comparison 1)

**Figure 2.**
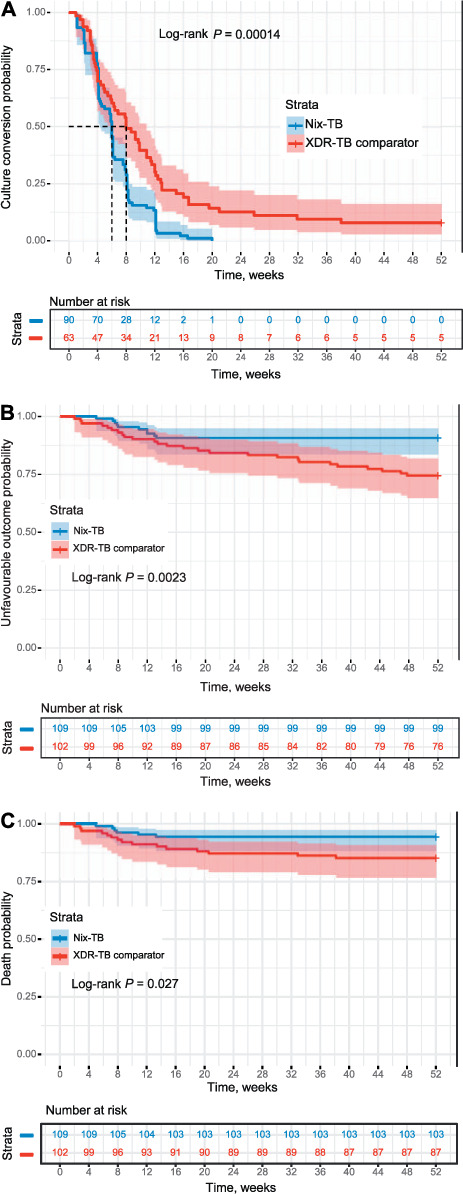
Kaplan–Meier curves depicting A) culture conversion probability: time to culture conversion for participants in the Nix-TB (n = 90) and the XDR-TB comparator cohort (n = 63) censored at 52 weeks;^*^ B) unfavourable outcome probability: time to unfavourable outcome in the total Nix-TB (n = 109) and the total XDR-TB comparator population (n = 102) censored at 52 weeks; and C) probability of death: time to death for all participants in the Nix-TB (n = 109) and XDR-TB comparator (n = 102) cohorts censored at 52 weeks. ^*^Only patients who were culture-positive at baseline were included in the analysis. Participants who died but did not culture convert were also included in this analysis. Solid lines represent time to event and the shaded areas represent the 95% CIs. XDR-TB = extensively drug-resistant TB; CI = confidence interval.

As shown in Table 2A and 2C, in the overall comparison between the groups the favourable outcome rate (%) was significantly better in those on the BPaL regimen than in the BDQ-based XDRTB cohort (*P* < 0.001; Table 2). Time to culture conversion (significantly more rapid with BPaL, *P* < 0.001), and time to unfavourable outcome and time to death (significantly longer with BPaL, *P* = 0.0023 and *P* = 0.027) are shown in Figures 2A, 2B and 2C, respectively.

**Table 2:** Comparison 1: overall outcome comparison between Nix-TB and XDR-TB comparator populations

	Nix-TB *n/N* (%)	XDR-TB comparator *n/N* (%)	*P* value^*^
**A)** Favourable vs. unfavourable outcomes			
Favourable	98/109 (89.9)	66/102 (64.7)	
Unfavourable	11/109 (10.1)	36/102 (35.3)	
Total	109	102	<0.01
**B)** Unfavourable outcomes			
Death	7/109 (6.4)	19/102 (18.6)	
Default	0/109 (0)	1/102 (0.9)	
Treatment failure	2/109 (1.8)	5/102 (4.9)	
Withdrawn	1/109 (0.9)	0/102 (0)	
Lost to follow-up	1/109 (0.9)	11/102 (10.7)	
Total	11/109 (10.1)	36/102 (35.3)	<0.01
**C)** Relative risk ratio for a favourable outcome in the Nix vs. the XDR-TB comparator populations			
	Nix-TB/XDR-TB Risk ratio (95% CI)		*P* value^*^

Unadjusted	1.39 (1.19–1.62)		<0.001
Adjusted^†^	1.35 (1.14–1.59)		<0.001

^*^ Derived from the likelihood ratio χ^2^ test.

^†^ Adjusted for sex, HIV status, age (years), weight (kg) and smear microscopy status.

XDR-TB = extensively drug-resistant TB; CI = confidence interval.

The demographic data for those who died in both cohorts are shown in Supplementary Table S4A. The overall relative risk ratio (RRR) for death in the Nix-TB vs. the XDR-TB comparator cohort is shown in Supplementary Table S4B and the RRR for death stratified by CD4 count is shown in Supplementary Table S4C.

#### Patients who received bedaquiline and linezolid (Comparison 2)

**Figure 1.**
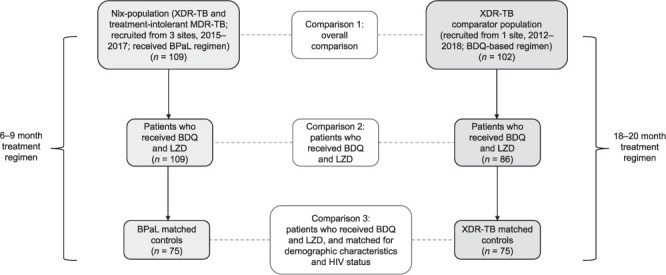
Study overview and analysis plan. XDR-TB = extensively drug-resistant TB; MDR-TB = multidrug-resistant TB; BPaL = BDQ, pretomanid and LZD; BDQ = bedaquiline; LZD = linezolid.

The Nix-TB cohort was also compared to the subgroup of participants from the XDR-TB comparator cohort who received BDQ and LZD as part of their treatment regimen (Comparison 2; Figure 1). As outlined in Table 3A and 3C, the Nix-TB population had a better favourable outcome (%) compared to this XDR-TB comparator subgroup (*P* < 0.001). The breakdown of unfavourable outcomes is shown in Table 3B.

**Table 3:** Comparison 2: outcome comparison between Nix-TB and XDR-TB comparator populations who received bedaquiline and linezolid

	Nix-TB *n/N* (%)	XDR-TB comparator *n/N* (%)	*P* value^*^
**A)** Favourable vs. unfavourable outcomes			
Favourable	98/109 (89.9)	56/86 (65.1)	
Unfavourable	11/109 (10.1)	30/86 (34.9)	
Total	109	86	<0.01
**B)** Unfavourable outcomes			
Death	7/109 (6.4)	15/86 (17.4)	
Default	0/109 (0)	1/86 (1.2)	
Treatment failure	2/109 (1.8)	4/86 (4.7)	
Withdrawn	1/109 (0.9)	0/86 (0)	
Lost to follow-up	1/109 (0.9)	9/86 (10.5)	
Total	11/109 (10.1)	29/86 (33.7)	<0.01
**C)** Relative risk ratio for a favourable outcome in the Nix vs. the XDR-TB comparator populations			
	Nix-TB/XDR-TB Risk ratio (95% CI)		*P* value^*^

Unadjusted	1.38 (1.17–1.63)		<0.001
Adjusted^†^	1.35 (1.13–1.60)		<0.001

^*^ Derived from the likelihood ratio χ^2^ test.

^†^ Adjusted for sex, HIV status, age (years), weight (kg) and smear microscopy status.

XDR-TB = extensively drug-resistant TB; CI = confidence interval.

#### Matched controls (Comparison 3)

In a further analysis, we compared those who received BDQ and LZD in the two population sub-samples individually matched by sex, age, body weight and HIV status at baseline (Figure 1). We aimed for a 1:1 Nix-TB to comparator ratio. Consequently, the two sub-samples included 75 Nix-TB participants and 75 matched controls. There were minimal differences between the two populations (Supplementary Table S1B).

As outlined in Table 4A, 4B and 4C the Nix-TB-population had a better favourable outcome (%) compared to the matched controls in the XDR-TB comparator population (*P* = 0.001).

**Table 4:** Comparison 3: outcome comparison between Nix-TB and XDR-TB comparator populations who received bedaquiline and linezolid, and individually matched for demographic data and HIV status

	Nix-TB *n/N* (%)	XDR-TB comparator *n/N* (%)	*P* value^*^
**A)** Favourable vs. unfavourable outcomes			
Favourable	67/75 (89.3)	48/75 (64.0)	
Unfavourable	8/75 (10.7)	27/75 (36.0)	
Total	75	75	<0.01
**B)** Unfavourable outcomes			
Death	4/75 (5.5)	15/75(20.0)	
Default	0/75 (0)	1/75 (1.3)	
Treatment failure	2/75 (2.7)	2/75 (2.7)	
Withdrawn	1/75(1.3)	0/75(0)	
Lost to follow-up	1/75 (1.3)	7/75 (12.0)	
Total	8/75 (10.7)	25/75 (33.3)	<0.01
**C)**			
	Nix-TB/XDR-TB Risk ratio (95% CI)		*P* value^*^

Unadjusted	1.40 (1.16, 1.68)		<0.001
Adjusted^†^	1.39 (1.14, 1.69)		0.001

^*^ Derived from the likelihood ratio χ^2^ test.

^†^ Adjusted for sex, HIV status, age (years), weight (kg) and smear microscopy status.

XDR-TB = extensively drug-resistant TB; CI = confidence interval.

When smear microscopy status was added to the matching criteria, the sample size decreased to 54 participants. The demographic data of this subgroup are shown in Supplementary Table S2 in the online supplement. As shown in Supplementary Table S3A and S3C, the Nix-TB-population showed a better favourable outcome (%) than matched controls in the XDR-TB comparator population (*P* = 0.037).

### HIV-infected vs. non-HIV-infected persons

The frequency of HIV infection (%) was similar for the Nix-TB cohort and the XDR-TB comparator cohort (56/109, 51.4% vs. 52/102, 51.0%). HIV-infected persons in the Nix-TB cohort had a better favourable outcome (%) than the XDR-TB comparator cohort (*P* = 0.011; Table 5A). A similar pattern was seen in non-HIV-infected persons (*P* = 0.007; Table 5B). The RRR stratified by CD4 count for those who were HIV-infected is shown in Table 5C.

**Table 5:** Outcome comparison of Nix-TB and XDR-TB comparator patients who were HIV-infected

		Nix-TB *n* (%)	XDR-TB comparator *n* (%)	*P* value^*^
**A)** HIV-infected				
Favourable		50 (89.3)	33 (63.5)	
Unfavourable		6 (10.7)	19 (36.5)	
Total		56	52	<0.01
		Nix-TB/comparator Risk ratio (95% CI)		*P* value^*^

Unadjusted		1.41 (1.12–1.76)		0.003
Adjusted^†^		1.35 (1.07–1.70)		0.011
**B)** Non-HIV-infected		Nix-TB *n* (%)	XDR-TB *n* (%) comparator	*P* value^*^

Favourable		48 (90.6)	33 (66.0)	
Unfavourable		5 (9.4)	17 (34.0)	
Total		53	50	<0.01
		Nix-TB/comparator Risk ratio (95% CI)		*P* value^*^

Unadjusted		1.37 (1.10–1.70)		0.004
Adjusted^†^		1.35 (1.08–1.67)		0.007
**C)** CD4, cells/mm^3‡^		Nix-TB/comparator Risk ratio (95% CI)		*P* value^*^

CD4 < 200	Unadjusted	1.47 (1.00–2.17)		0.05
CD4 < 200	Adjusted^†^	1.37 (0.91–2.08)		0.13
CD4 > 200	Unadjusted	1.36 (0.96–1.91)		0.079
CD4 > 200	Adjusted^†^	1.24 (0.85–1.80)		0.27

^*^ Derived from the likelihood ratio χ^2^ test.

^†^ Adjusted for sex, age (years), weight (kg) and smear microscopy status.

^‡^ Due to the small number of participants with a CD4 count <200 cells/μL (seven in the Nix-TB population and only one with an unfavourable outcome in the same population), we were unable to meaningfully adjust for CD4 count or perform further analyses.

XDR-TB = extensively drug-resistant TB; CI = confidence interval.

## DISCUSSION

We performed a cohort-comparison study with prospective follow-up comparing patients with XDR-TB and treatment intolerant MDR-TB who received the 6- to 9-month BPaL regimen (starting dose of 1200 mg LZD daily) to patients with XDR-TB who received an 18- to 20-month 8-drug (median) B–L-based regimen (starting dose of 600 mg LZD daily). The key finding was that the patients receiving the BPaL regimen had a significantly better 6-month post-treatment outcome than those on the B–L-based regimen when controls were matched for demographic characteristics and HIV status, and outcomes were adjusted for smear-related mycobacterial disease burden. This was the case even though the Nix-TB cohort included a significant proportion of DR-TB treatment failures with a significantly higher rate of prior second-line drug exposure. Nix-TB patients also received a median of five fewer drugs than the XDR-TB comparator cohort. Thus, although the baseline characteristics of the cohorts were not entirely concordant (which we acknowledge and discuss in the limitations section below), the Nix-TB cohort likely had worse prognostic features but potentially better outcomes. The outcome benefit of BPaL was seen in both HIV-infected and non-infected persons (the effect of CD4 count is discussed below). Treatment outcome comparisons were made at 6 months after treatment completion in the Nix-TB population and up to 24 months post-treatment initiation in the comparator cohort. Despite the difference in assessment timing relative to treatment initiation, 12-month post-treatment initiation outcomes have been shown to correlate well with longer-term outcomes and even 6-month culture positivity rates correlate highly with unfavourable outcomes.^4^,^16^,^17^ Indeed, it appears that even at 24 months post-treatment completion, the outcome benefit was sustained.

The outcome benefit with the Nix-TB regimen was seen despite 1) the shorter duration of treatment (~6 months vs. 18–20-month BDQ-LZD regimen) in patients with XDR-TB; 2) the fact that most patients in the Nix-TB trial were on prior treatment with second-line drugs; and that 3) on average, five fewer drugs compared to the XDR-TB comparator cohort were administered. The outcome benefit of the Nix-TB regimen was sustained even after adjustment for prognostic characteristics such as HIV status (CD4 is discussed below under limitations), age, weight and mycobacterial disease burden (smear status that was differentially distributed between the groups and adjusted for in the analysis), etc. Indeed, PTM showed good early bactericidal activity (EBA) in two-phase 2A studies and murine studies, indicating that BPaL performed better than any two-drug combinations of BDQ, LZD and/or PTM.^12^ The starting LZD dose of 1200 mg may have added to the efficacy of the regimen, as the EBA-associated mycobactericidal effect of LZD was shown to be dose-dependent in drug-susceptible TB.^18^ A 1200 mg daily LZD dose may be associated with better outcomes, and this dose is also likely to have the best effect in terms of preventing resistance amplification based on pharmacokinetic studies (given the higher bactericidal activity).^12^,^19^ Nevertheless, drug efficacy is likely to be related to a number of factors, including population-level heterogeneity in AUC/MIC (area under the curve/minimum inhibitory concentration) targets, host-related factors (absorption, metabolism, genetics, etc.) and intracavitory and lesion-specific drug penetration, among others. There is also possible synergistic activity between BDQ, PTM and LZD.^12^,^20^,^21^

While these are the first published comparative data of BPaL vs. a B-L-containing regimen in patients with DR-TB, there are a number of implications and limitations to our study. 1) The prospective Nix-TB study was not a controlled one and the cohorts being compared were recruited at time periods that did not fully overlap, and in a different context (one was a prospective cohort study, the other was a prospective Phase 3 registration study performed under strict Good Clinical Practice conditions). The sample size was also limited. However, both cohorts were prospectively recruited, and both recruited HIV-infected and non-infected patients. Both were performed in the same geographical setting, i.e., South Africa (and many from the same facility in Cape Town), and the sample size was large enough to demonstrate significant differences in regimen efficacy. Furthermore, the efficacy benefit was seen even when matched controls (matched for BDQ and LZD usage, demographic characteristics and HIV status) were compared, and when the findings were adjusted for prognostic characteristics such as weight, HIV status and baseline bacterial load. Thus, although the baseline characteristics had some differences (further matching would have substantially reduced sample size and hence, power), we adjusted our analysis for these differences as best as we could. Indeed, we performed a separate analysis matching for smear status in a smaller group of 54 paired patients (Supplementary Table S2), which showed the same pattern of outcome benefit. 2) Related to this latter point, median CD4 count was significantly lower in HIV-infected persons in the XDR-TB group, thus potentially confounding our results. However, we could not perform propensity matching for this characteristic, given the effect on the sample size (matching would have produced 28 matched pairs, severely reducing power), and we could not adjust for CD4 in the HIV outcome analysis (Table 5) because of limited power (only one person in the Nix-TB cohort had an unfavourable outcome with a CD4 < 200 cells/mm^3^). However, we did further stratify analysis by CD4 count (Table 5), and if the adjusted risk ratio in those with advanced immunosuppression was a major driver, we would have likely seen a significant difference in this stratum (to note, the effect we see is in the opposite direction, i.e., in favour of Nix despite the higher frequency of advanced immunosuppression in the XDR-TB group). 3) The study findings may not be generalisable to other settings where HIV is not endemic and the strain heterogeneity is different due to the abovementioned drawbacks. 4) Both regimens were of different durations (6 months vs. 18–24 months of intended treatment), and the primary endpoint was at 6 months post-treatment completion for the Nix-TB cohort and at 24 months post-treatment initiation for the XDR-TB comparator cohort. To note, outcome data were assessed, on average, at 6 months post-treatment completion in both groups. However, both 6- and 12-month outcomes have been shown to correlate very well with long-term outcomes in patients with XDR-TB.^4^,^7^ Indeed, upon reviewing 47 Nix-TB participants who were enrolled and had the opportunity to reach the 24-month post-treatment completion time-point, only one additional unfavourable outcome, a relapse, has been recorded. This degree of follow-up after treatment is greater than that of the comparator group. Furthermore, the outcome benefits seen with the BPaL regimen is even more striking, given that the treatment regimen was of only 6 months’ duration in all but two patients. Another aspect related to the regimen is that comparative BDQ and LZD susceptibility testing was not performed; however, standardised assays were not programmatically available at the time, and we showed that background resistance to LZD and BDQ was extremely low in this population.^1^ Furthermore, there may have been differential susceptibility to oflaxacin vs. levofloxacin, which may have impacted outcomes. 5) The potentially better outcomes seen in the BPaL regimen may be partly or completely due to the Hawthorne effect, given that this was performed under strict research study conditions.^22^ Indeed, in the STREAM (Standard Treatment Regimen of Anti-tuberculosis Drugs for Patients With MDR-TB) I study the favourable outcome rate for MDR-TB patients even with a non-BDQ and non-LZD injectable-based regimen was as high as 83%.^23^ 6) The possibility of a survival bias effect cannot be ruled out, i.e., Nix-TB self-selected for survivors and patients with the most severe disease were not included. However, Nix-TB enrolled patients with poor prognostic features (those who had failed MDR- or XDR-TB treatment), and the same bias applied to the XDR-TB comparator group, as only those initiating treatment were included in the cohort. 7) We did not perform safety comparisons between the groups. However, drug toxicity profiles are reported in the parent manuscripts,^4^,^11^ and different intensities of safety monitoring and significant observation bias precluded comparison of adverse events and laboratory abnormalities; nevertheless, safety issues that were fatal or led to discontinuation are incorporated in the outcome measures. Finally, it is possible that the outcome effect seen could have been influenced by disease extent (extensive parenchymal disease and cavitation), prior drug treatment exposure, and differential culture negativity at baseline. However, demographic baseline characteristics were similar, and we adjusted for weight and baseline bacterial burden (an important prognostic feature) in the risk ratio analysis. Furthermore, we compared comparative drivers of mortality in the cohorts (see online Supplementary Data) and accounted for CD4 count differences in the HIV analysis (as outlined above) given that the level of immunosuppression was higher in the XDR-TB comparator group.

In conclusion, in this cohort-comparison study with prospective follow-up, the ~6-month BPaL regimen showed potentially better 6-month post-treatment outcomes in patients with XDR-TB compared to an ~18-month B–L-based regimen. These data, despite the limitations noted, may lend support to the conclusion that PTM likely plays a significant contributory role to the efficacy seen with the BPaL regimen. However, given the limitations of the current analysis, further controlled studies are required to definitively answer the question.
